# Deglobalisierung, Rekonfiguration oder Business as Usual? COVID-19 und die Grenzen der Rückverlagerung globalisierter Produktion

**DOI:** 10.1007/s11609-022-00479-5

**Published:** 2022-09-01

**Authors:** Florian Butollo, Cornelia Staritz

**Affiliations:** 1 Weizenbaum-Institut für die vernetzte Gesellschaft, Wissenschaftszentrum Berlin für Sozialforschung, Hardenbergstr. 32, 10623 Berlin, Deutschland; 2 Institut für Internationale Entwicklung, Universität Wien, Sensengasse 3, 1090 Wien, Österreich

**Keywords:** COVID-19-Pandemie, Globale Produktionsnetzwerke, Re‑/Nearshoring, Deglobalisierung, Geopolitik, Automobil, Bekleidung, Elektronik, COVID-19 pandemic, Global production networks, Re‑/Nearshoring, Deglobalization, Geopolitics, Automotive, Clothing, Electronics, Pandémie du COVID-19, Réseaux de production mondiaux, Re‑/Nearshoring, Déglobalisation, Géopolitique, Automobile, Habillement, Électronique

## Abstract

Die wirtschaftlichen Erschütterungen infolge der COVID-19-Pandemie scheinen die Notwendigkeit einer geografischen Restrukturierung und Rückverlagerung der Produktion zu bestärken, führten sie doch die Verwundbarkeit der globalisierten Produktionsstrukturen vor Augen. Der Beitrag geht den Auswirkungen von COVID-19 auf die Geografie globaler Produktionsnetzwerke nach. In Abgrenzung zu unterkomplexen Perspektiven auf die Globalisierung wird deren multiskalarer und politisch gestalteter Charakter hervorgehoben. Basierend auf diesen theoretischen Überlegungen und Fallstudien zur Automobil‑, Elektronik- und Bekleidungsindustrie wird gefolgert, dass die COVID-19-Pandemie nicht als Auslöser für einen allgemeinen Rückbau der globalen Fertigung interpretiert werden kann, wohl aber bereits länger anhaltende Verschiebungen hin zu multipolaren Produktions- und Konsumstrukturen verstärkt. Zwar hat das Thema der Resilienz globaler Produktionsnetzwerke eine größere Aufmerksamkeit in der strategischen Planung von Unternehmen und der Industriepolitik von Staaten erhalten. Eine verstärkte Lokalisierung und Regionalisierung von Produktionsnetzwerken ist jedoch nur eine Strategie von mehreren und wurde bis jetzt kaum implementiert. Anhaltende Störungen der Lieferketten, steigende Transportkosten und vor allem geo- und umweltpolitisch motivierte Politiken könnten aber durchaus zu einem stärkeren Re- oder Nearshoring führen. Politische Bestrebungen in diese Richtung werden jedoch limitiert durch gewachsene weltwirtschaftliche Entwicklungspfade und die mit ihnen verbundenen Kräfteverhältnisse. Im Ausblick betont der Beitrag die Notwendigkeit einer umfassenden politisch motivierten Restrukturierung globaler Produktionsnetzwerke im Kontext der dringend gebotenen sozial-ökologischen Transformation.

## Abstract (en)

The economic difficulties following the COVID-19 pandemic have seemingly reinforced the need for geographic restructuring and a reshoring of production, as they have demonstrated the vulnerability of globalized production. This article provides an assessment of the impact of COVID-19 on the geographies of production. Criticizing overly simplified perspectives on globalization, the article argues that global production networks are multiscalar and politically shaped phenomena. Based on these theoretical considerations and case studies on the automotive, electronics and clothing industries, the article concludes that the COVID-19 pandemic cannot be interpreted as a trigger for a general retreat from global manufacturing, but it reinforced longer-standing shifts toward more multipolar production and consumption structures. While the issue of global production network resilience has attracted greater attention in corporate strategies and industrial policies, the localization and regionalization of production networks is only one of several strategies, and it has hardly been implemented so far. Ongoing disruptions of supply chains, increased transport costs, and, above all, geopolitically and environmentally motivated policies could well lead to greater re- or nearshoring. Political efforts in this direction are, however, limited by pre-existing global economic development paths and the balance of power associated with them. In the conclusions, the article stresses the necessity of a politically motivated restructuring of global production networks in the context of an urgently needed social-ecological transformation.

## Résumé

Les secousses économiques provoquées par la pandémie COVID-19 semblent avoir renforcé la nécessité d’une restructuration géographique et d’une relocalisation de la production industrielle, tout en mettant en évidence la vulnérabilité des structures de production mondialisées. L’article examine les effets de COVID-19 sur la géographie des réseaux de production mondiaux et met en évidence son caractère multiscalaire et politiquement structuré, ainsi se distinguant des perspectives sous-complexes de la mondialisation. Sur la base de ces réflexions théoriques et d’études de cas sur les industries de l’automobile, de l’électronique et de l’habillement, il conclue que la pandémie de COVID-19 ne peut pas être interprétée comme le déclencheur d’un démantèlement généralisé de la production mondiale, mais qu’elle renforce des déplacements déjà anciens vers des structures de production et de consommation multipolaires. La question de la résilience des réseaux de production mondiaux a certes fait l’objet d’une attention accrue dans la planification stratégique des entreprises et la politique industrielle des États. Cependant, une localisation et une régionalisation accrues des réseaux de production ne sont qu’une stratégie parmi d’autres et n’ont guère été mises en œuvre jusqu’à présent. Les perturbations persistantes des chaînes d’approvisionnement, l’augmentation des coûts de transport et surtout les politiques géopolitiques et environnementales pourraient toutefois tout à fait conduire à un « re- » ou « nearshoring » plus important. Les efforts politiques dans ce sens sont toutefois limités par les voies de développement de l’économie mondiale et les rapports de force qui y sont liés. En conclusion, l’article souligne la nécessité d’une restructuration globale des réseaux de production mondiaux, motivée par des raisons politiques, dans le contexte d’une transformation socio-écologique urgente.

## Einleitung

Die wirtschaftlichen Erschütterungen infolge der COVID-19-Pandemie werfen die Frage nach der Zukunftsfähigkeit der derzeitigen wirtschaftlichen Ordnung auf. Das gilt insbesondere für ihre weltumspannende geografische Struktur. Schon seit der Finanz- und Wirtschaftskrise 2008/09 findet sich die Einschätzung, dass die Globalisierung der Produktion ihren Zenit überschritten habe. Der „Economist“ prägte angesichts der Verlangsamung von Globalisierungstendenzen den Begriff „Slowbalization“ (The Economist 2019), und von unterschiedlicher Seite werden unter den Begriffen „Reshoring“, „Backshoring“ oder „Nearshoring“ Erwartungen einer Rückverlagerung von Fertigungskapazitäten in Länder des Globalen Nordens artikuliert. Als Treiber solcher Tendenzen werden die Störungsanfälligkeit globaler Produktionsnetzwerke aufgrund von Naturkatastrophen bzw. menschengemachter Schocks, Verschiebungen in der Lohnkostenstruktur, die Wirkungen der Digitalisierung, die zunehmenden Spannungen in der Handelspolitik durch geopolitische und -ökonomische Verschiebungen sowie klimapolitische Vorgaben und Ziele identifiziert. Die geografische Restrukturierung kann also markt- oder politikgetriebene Ursachen haben, wobei in der Literatur diesen Ursachen ein jeweils unterschiedliches Gewicht beigemessen wird (Javorcik 2020; Kinkel 2020; Lund et al. 2020; Raza et al. 2021; Shih 2020; UNCTAD 2021).

Die COVID-19-Pandemie scheint die Notwendigkeit einer geografischen Restrukturierung von Produktionsnetzwerken zu bestärken, führte sie doch die Verwundbarkeit der globalen Just-in-time-Produktion vor Augen (Haass 2020; Irwin 2020). Der chinesische Lockdown ab Januar 2020 führte schnell zu Lieferausfällen bei wichtigen Vor- und Endprodukten für die industrielle Produktion. Engpässe bei der Versorgung mit medizinischen Produkten zur Pandemiebekämpfung steigerten die Besorgnis über eine übermäßige Abhängigkeit von globalen Importen bzw. einer Aushöhlung industrieller Kapazitäten in der EU und den USA. Viele Lieferketten, z. B. im Bereich von Möbeln, Schuhen oder Elektronikprodukten, blieben auch im Jahr 2021 störanfällig, infolge von weiteren Lockdowns und anhaltenden Problemen im Seefrachtverkehr sowie einer zugleich deutlich angestiegenen Nachfrage. Infolgedessen wurde vielfach die Erwartung formuliert, dass diese Erfahrungen in den Unternehmen zu einem Umdenken führen müssten, um eine größere Resilienz zu erreichen: durch eine Reduktion der Abhängigkeit von den globalen und insbesondere chinesischen Zulieferfirmen und eine Stärkung von regionalen Produktionsnetzwerken.

Der vorliegende Beitrag geht den Auswirkungen von COVID-19 auf die Geografie von globalen Produktionsnetzwerken nach. Im Mittelpunkt steht die Frage, ob die Pandemie als Auslöser für Rückverlagerungen und eine Deglobalisierung von globalen Fertigungsstrukturen angesehen werden kann, wobei wir besonders auf Entwicklungen in der Automobil‑, Elektronik- und Bekleidungsindustrie eingehen. Ausgangspunkt ist die Kritik an der theoretisch verkürzten Debatte um Reshoring und Deglobalisierung. Gegenüber einer reduktionistischen Gegenüberstellung von Off- und Re‑/Nearshoring stellen wir heraus, dass wirtschaftliche Globalisierung und globale Produktionsnetzwerke multiskalare und dynamische Phänomene sind, bei denen globale Auslagerungen, regionale Produktionscluster und lokal konzentrierte Operationen eng miteinander verbunden sind. Die derzeitige Ausgestaltung globaler wie regionaler Produktionsnetzwerke basiert auf dem auf kurzfristige Effizienz ausgelegten Rationalisierungsparadigma flexibler Fertigung. Leitunternehmen minimieren so die Kosten von Lagerhaltung und Redundanzen und maximieren Flexibilisierung und Beschleunigung der Lieferketten, was Zulieferfirmen und deren Arbeiter*innen unter Druck setzt. Globale Produktionsnetzwerke sind zugleich politisch gestaltete Phänomene. Unternehmensstrategien sind in Welthandelsregime und Industriepolitiken eingebettet, die im Kontext von geopolitischen und -ökonomischen Spannungen sowie zunehmenden Ambitionen (grüner) Industriepolitik einem Wandel unterlegen sind.

Die These unseres Beitrags ist daher, dass die COVID-19-Pandemie nicht als Auslöser für einen allgemeinen Rückbau globaler Fertigung oder gar einer Deglobalisierung interpretiert werden kann, wohl aber schon länger anhaltende Verschiebungen hin zu stärker multipolaren Produktions- und Konsumstrukturen verstärkt. Zwar hat das Thema der Resilienz globaler Produktionsnetzwerke eine größere Aufmerksamkeit in der strategischen Planung von Unternehmen und der Industriepolitik von Staaten erhalten. Eine verstärkte Regionalisierung von Produktionsnetzwerken ist allerdings nur eine Strategie von mehreren und bis jetzt kaum empirisch nachzuweisen. Anhaltende Störungen der Lieferketten, steigende Transportkosten und vor allem handelspolitische Spannungen sowie geoökonomisch bzw. geopolitisch und umweltpolitisch motivierte Politiken könnten mittelfristig durchaus zu einem stärkeren Re- oder Nearshoring bzw. einer noch stärkeren regionalen Blockbildung führen. Während der Pandemie spitzte sich der Konflikt um die Konturen des Welthandels und die Bedeutung strategischer Industriepolitik zu, da Fragen der Versorgungssicherheit und der technologischen Souveränität in den Vordergrund rückten. Die (Post‑)COVID-19-Phase wird also wesentlich von politischen Zielsetzungen geprägt, die sich auf die geografische Struktur der Produktion niederschlagen können. Allerdings werden diese politischen Bestrebungen limitiert durch die anhaltende Hegemonie des Rationalisierungsparadigmas der flexiblen Fertigung, gewachsene weltwirtschaftliche Entwicklungspfade und die mit ihnen verbundenen Kräfteverhältnisse, was eine umfassende Deglobalisierung sehr unwahrscheinlich macht. Der russische Angriff auf die Ukraine im Februar 2022, in dessen Folge sich die geopolitischen Spannungen weiter zuspitzen, dürfte allerdings ein weiterer Treiber einer strategischen Neuausrichtung in den Geografien der Produktion und des Handels sein – weniger im Sinne eines Rückbaus der Strukturen, denn einer Blockbildung im internationalen Produktions- und Handelsregime. Die Effekte dieses historischen Einschnitts können in diesem Beitrag jedoch nicht systematisch erfasst und ausgewertet werden, zumal sie derzeit noch kaum absehbar sind.

Wir stützen uns in unserer nachfolgenden Analyse auf drei Fallstudien zu Produktionsnetzwerken in der Automobil‑, Elektronik- und Bekleidungsindustrie, weil besonders diese Sektoren in den letzten Jahrzehnten von einer Globalisierung der Produktion gekennzeichnet waren. Methodisch bauen die Studien auf der Auswertung von Sekundärliteratur und der Analyse aktueller Quellen zu geografischen Restrukturierungen im Kontext der COVID-19-Pandemie auf. Diese Quellen umfassen die Branchenberichterstattung in einschlägigen Zeitschriften, Portalen und Konferenzen oder Roundtables. Des Weiteren wurden insgesamt acht semi-strukturierte Interviews mit Industrieexpert*innen sowie Industrieverbands- und Unternehmensvertreter*innen aus zwei der drei Sektoren geführt.^1^ Das Material aus den Quellen wurde zu kurzen Synopsen über die drei untersuchten Sektoren verdichtet. Im Ergebnis stellt unsere Untersuchung eine theoretisch informierte Momentaufnahme dar, die Tendenzen und Entwicklungsoptionen beschreibt, jedoch keine endgültigen Befunde zu den Entwicklungen liefern kann, die großenteils noch im Fluss und angesichts zunehmender geopolitischer – und seit dem russischen Angriff auf die Ukraine auch kriegerischer – Konflikte kontingent sind. Durch die Einbeziehung aktueller Einschätzungen von Industrieexpert*innen aus Wissenschaft und Praxis konnten jedoch fundierte Einblicke in aktuelle Debatten, Restrukturierungen und mögliche Verschiebungen gewonnen werden.

Im nachfolgenden Abschnitt 2 skizzieren wir zunächst eine theoretische Perspektive auf wirtschaftliche Globalisierung als ein multiskalares, dynamisches und politisch gestaltetes Phänomen; dieses Verständnis liegt unserer Untersuchung möglicher Verschiebungen in der Geografie globaler Produktionsnetzwerke zugrunde. Diese beginnt mit der Darstellung von geografischen Verschiebungen seit der globalen Finanz- und Wirtschaftskrise 2008/09 (Abschn. 3) sowie einer kritischen Diskussion der in der Literatur genannten ökonomischen und politischen Treiber dieser Verschiebungen (Abschn. 4). Die Interpretation dieser schon länger anhaltenden Verschiebungen ist zentral für ein Verständnis der Entwicklungen im Kontext der COVID-19-Pandemie, die wir im Anschluss diskutieren (Abschn. 5). Wir geben einen Überblick über den Einbruch von globalen Produktionsnetzwerken während der Pandemie sowie über die Ursachen von anhaltenden Störungen der Lieferketten und diskutieren, inwieweit sich im Zuge der COVID-19-Pandemie eine qualitative Veränderung der geografischen Struktur der Wertschöpfung andeutet (Abschn. 6). Eine Analyse der jüngeren Entwicklungen in der Automobil‑, Elektronik- und Bekleidungsindustrie (Abschn. 7) fundiert die Einschätzung, dass eine verstärkte Regionalisierung von Produktionsnetzwerken nur eine unter mehreren Strategien und bisher empirisch kaum nachzuweisen ist. In einem Ausblick (Abschn. 8) betonen wir die Notwendigkeit einer umfassenden politisch motivierten Restrukturierung globaler Produktionsnetzwerke im Kontext der dringend gebotenen sozial-ökologischen Transformation.

## Globalisierung als multiskalares und politisch gestaltetes Phänomen

Die Diskussion um Deglobalisierung zeichnet oft ein bipolares Bild globaler Produktion, das auf einer reduktionistischen Global-lokal-Dichotomie und einer simplen Gegenüberstellung von Off- und Re‑/Nearshoring beruht. Diese Sichtweise unterschlägt, dass globale Produktionsnetzwerke dynamisch und multiskalar sind, d.h. unterschiedliche geografische Skalen und Reichweiten von Produktionsprozessen (lokal, regional, national, global) in Netzwerken integrieren, die einem steten Wandel unterliegen. Obwohl in vielen Produktionsnetzwerken Produkte von globalen Zulieferfirmen, insbesondere aus Ländern des Globalen Südens, bezogen werden, hat die globale Produktion immer auch eine lokale und regionale Dimension, wobei globale Auslagerungen, regionale Produktionscluster und lokal konzentrierte Operationen eng miteinander verbunden sind. Auch bei den am stärksten globalisierten Sektoren wie der Bekleidungs- oder Elektronikindustrie spielen regionale Zulieferer und die Konzentration der Produktion in regionalen Clustern eine wichtige Rolle. Die Automobilindustrie ist noch stärker regional rund um zentrale Endmärkte organisiert. Einige prozessorientierte Branchen wie z. B. die Metallteile‑, Papier- und Zementindustrie operieren sogar primär intraregional (Lund et al. 2019, S. 27 ff.). Allgemein gesprochen, ist die Regionalisierung nicht zwangsläufig ein gegensätzlicher Trend zur Globalisierung; Prozesse der regionalen Konzentration von Produktion können etwa innerhalb der Logiken globaler Produktionsnetzwerke stattfinden.

Die derzeitige Ausgestaltung globaler wie regionaler Produktionsnetzwerke basiert auf einer Managementorientierung, die auf kurzfristige Effizienzgewinne und die Just-in-time-Produktion setzt. Dies führt zu einer Reduktion von Lagerbeständen und Redundanzen sowie einer fortschreitenden Rationalisierung, Flexibilisierung und Beschleunigung der Lieferketten. Dieses, wie weiter unten argumentiert, weiterhin hegemoniale Rationalisierungsparadigma der flexiblen Fertigung geht einher mit der Auslagerung von Kosten und Risiken von sogenannten Leitunternehmen an Zulieferfirmen, die den Druck oft an ihre Arbeiter*innen weitergeben. Sogar bei den klassischen arbeitsintensiven Industrien wie etwa dem Bekleidungssektor spielen neben den Arbeits- und anderen direkten und indirekten Produktionskosten auch Faktoren wie Qualität, Geschwindigkeit, Flexibilität und die Kapazitäten von Zulieferfirmen, weitere Aufgaben wie Lagerhaltung und Finanzierung für Leitunternehmen zu übernehmen, eine zentrale Rolle bei Sourcing-Entscheidungen (Palpacuer et al. 2005). Innovationsintensive Branchen wie die Chemie‑, Automobil- und Elektronikindustrie folgen bei ihren Investitions- und Sourcing-Entscheidungen stärker auch anderen Prioritäten als der reinen (Arbeits‑)Kosteneinsparung. Dazu gehört die jeweilige Verankerung in komplexen Ökosystemen und Clustern aus Forschung & Entwicklung und Fertigungsoperationen, wobei für die Leitunternehmen die sehr profitable Kombination von „high tech“ und „low wages“ am vorteilhaftesten ist (Baldwin 2016).

Darüber hinaus zielen die Investitions- und Vergabeentscheidungen von Leitunternehmen in vielen Sektoren nicht nur auf für sie vorteilhafte Produktionsbedingungen, sondern auch auf Marktnähe und Marktzugang. Diese Tendenz hat sich durch die gestiegene Bedeutung von Märkten in den großen Ländern des Globalen Südens weiter verstärkt (Herrigel 2015; ten Brink und Nölke 2013). Nicht nur Produktions-, sondern auch Innovationsprozesse differenzieren sich dadurch zunehmend zu einer multipolaren Struktur aus. Dies bedeutet auch, dass Nearshoring und Regionalisierung nicht allein mit Rückverlagerungen von Produktionseinheiten nach Europa oder in die USA gleichzusetzen sind. Denn Nearshoring kann auch dadurch entstehen, dass europäische oder amerikanische Firmen Investitionen in räumlicher Nähe zu asiatischen Endmärkten oder Produktionsclustern tätigen.

Eine fundierte Einschätzung der Aussichten einer möglichen Rückverlagerung der Produktion lässt sich somit nur treffen, wenn man sich die multiskalare und dynamische Gestalt von globalen Produktionsprozessen vergegenwärtigt. Die Organisation und Governance von globalen Industrien und ihre geografische Ausgestaltung, die Formen der Wertschöpfung und -aneignung sowie die Verteilung von Kosten und Risiken hängen zentral von den Strategien und Praktiken von Leitunternehmen ab, die als die primären „organising agents“ des globalen Kapitalismus verstanden werden können (Gereffi 1994, S. 97). Eine unternehmenszentrierte Perspektive muss jedoch die Einbettung dieser Akteure in sozialräumliche Kontexte thematisieren und dabei insbesondere ihre politische Strukturiertheit in den Blick nehmen (Henderson et al. 2002). Die starke Verbreitung globaler Produktionsnetzwerke seit den 1970er- und vor allem den 1990er- und 2000er-Jahren basiert beispielsweise nicht nur auf technologischen Fortschritten im Transport und der Informations- und Kommunikationstechnologie, sondern auch auf politischen Weichenstellungen und Bemühungen, einen globalen Wirtschaftsraum mit möglichst einheitlichen Regeln zu schaffen, der durch die Welthandelsorganisation sowie bilaterale und regionale Handels- und Investitionsabkommen abgesichert wurde (Linsi 2021; Raza 2020a).

Staatliche Regulierungen und Politiken sind also zentrale Voraussetzungen für die derzeitige globale Ausgestaltung von Produktion. Seit der Finanz- und Wirtschaftskrise 2008/09 hat die Bedeutung von strategischer Industriepolitik im Kontext von geopolitischen und -ökonomischen Konflikten um internationale Wettbewerbsfähigkeit und technologische Führerschaft und Souveränität zugenommen (ten Brink und Nölke 2013). Während der COVID-19-Pandemie rückte zudem die Notwendigkeit staatlicher Interventionen zur Stabilisierung der Versorgungssicherheit ins Zentrum politischer Aufmerksamkeit, und angesichts von virulenten Lieferkettenunterbrechungen wurden Rufe nach einer größeren Autarkie von globalen Lieferbeziehungen laut. Die Handlungsautonomie von Staaten ist jedoch eingeschränkt und von gesellschaftlichen sowie internationalen Kräfteverhältnissen geprägt (Linsi 2021; Raza et al. 2021). Schließlich können auch nicht-intendierte Folgen politischen Handelns die Steuerungsfähigkeit des Staates zusätzlich beeinträchtigen. Bestimmte Handels- und Industriepolitiken, wie zuletzt die wechselseitigen Handelsrestriktionen zwischen den USA und China, führen beispielsweise zu schwer prognostizierbaren Reaktionen von Unternehmen, die den Zielen der politischen Akteure zuwiderlaufen können (Gereffi et al. 2021).

## Globalisierungsdämmerung vor COVID-19?

**Figure 1:**
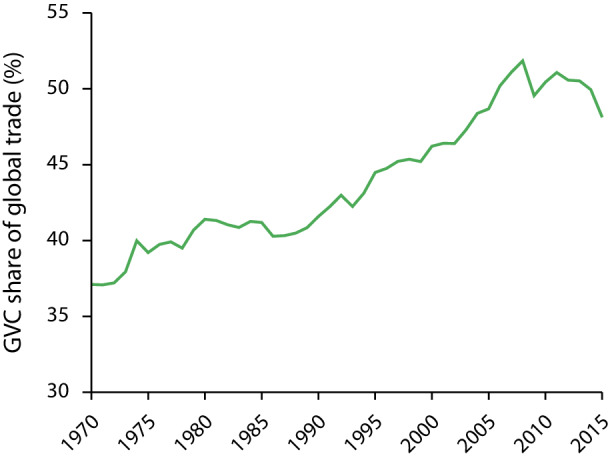

Die jüngsten Erschütterungen globaler Lieferketten schließen an Veränderungen an, die bereits vor der Pandemie als ein Ende oder ein Rückbau der Globalisierung gedeutet wurden (Cattaneo et al. 2010). Während seit der Finanz- und Wirtschaftskrise 2008/09 tatsächlich Veränderungen in der Geografie der Produktion stattfanden, wird das Ausmaß einer Deglobalisierung oft überzeichnet. Unstrittig ist, dass sich die Expansion globaler Produktionsnetzwerke im letzten Jahrzehnt verlangsamte. Trotz Erholung der globalen Ökonomie nach der Finanz- und Wirtschaftskrise erreichte das internationale Handels- und Investitionswachstum nicht das gleiche Ausmaß wie vor der Krise, und auch der Handel in globalen Produktionsnetzwerken (statistisch definiert als Handel mit Gütern, die mindestens zweimal eine Grenze überschreiten) stagniert seitdem auf einem Niveau von um die 50 % (World Bank 2020); 2015 lag der Wert um etwa vier Prozentpunkte unter dem Höchstwert von 52 % im Jahr 2008 (vgl. Abbildung 1).

Diese Verschiebungen haben zentral mit der veränderten Rolle Chinas und anderer Schwellenländer in globalen Produktionsnetzwerken zu tun und gehen nicht auf einen Rückbau oder eine Rückverlagerung der dortigen Produktionskapazitäten zurück. Der Anteil der traditionellen weltwirtschaftlichen Zentren (EU, USA, Japan) an der Wertschöpfung in globalen Produktionsnetzwerken für Industriegüter hat zwischen 2000 und 2014 von 60 % auf 44 % abgenommen. China hat im selben Zeitraum seinen Anteil von ca. 5 % auf 20 % vervierfacht (Turégano und Marschinski 2020). Während China nach der Krise 2008/09 weiter ein Knotenpunkt globaler Produktionsnetzwerke blieb, gibt es mittlerweile eine verstärkte Binnenzentrierung des chinesischen Wachstumsmodells. Dies ist eine Folge des schnell wachsenden Binnenkonsums sowie einer forcierten Orientierung des chinesischen Staates auf eine größere wirtschaftliche Eigenständigkeit, was sich unter anderem in industriellen Aufwertungsstrategien wie der Digitalstrategie „Made in China 2025“ und den sich daran anschließenden Programmen zur Förderung Künstlicher Intelligenz und des industriellen Internets äußert (Butollo und Lüthje 2017; Schmalz 2018).

Die Regionalisierung spielt nicht nur in Bezug auf China eine wichtigere Rolle. Seit Beginn der 2010er-Jahre hat der Anteil des intraregionalen Handels am gesamten Welthandel wieder zugenommen – vom niedrigsten Wert von 45 % in den Jahren 2012 und 2013 auf fast 48 % im Jahr 2017 (Lund et al. 2019, S. 38 f.). In einigen Regionen spielt der regionale Handel eine noch bedeutendere Rolle: Bei einem durchschnittlichen europäischen Land stammten beispielsweise im Jahr 2018 etwa 65 % der in den Exporten verwendeten importierten Vorleistungen aus einem anderen europäischen Land. In Ostasien und im Pazifik liegt der Anteil des intraregionalen Handels bei 55 %, während Nordamerika (39 %), Lateinamerika und die Karibik (26 %) sowie Subsahara-Afrika (11 %) deutlich geringere regionale Integrationsgrade aufweisen (World Bank 2020, S. 24 f.). Der amerikanisch-chinesische Handelskonflikt verstärkte den intraregionalen Handel innerhalb dieser beiden Blöcke weiter. Eine vollständige Entkopplung der Produktionsnetzwerke ist aber auch für China und die USA, wie im Folgenden noch ausgeführt wird, sehr unwahrscheinlich.

Insgesamt haben wir es also mit einer Neukonfigurierung des Zusammenspiels von globalen und regionalen Verbindungen in Produktionsnetzwerken zu tun. Nach der Phase der Hyperglobalisierung in den 1990er- und 2000er-Jahren kam es zu einer „Slowbalization“ und stärkeren Konsolidierung von einigen Produktionsschritten um regionale Knotenpunkte und zur Ausdifferenzierung einer stärker multipolaren Weltwirtschaft, vor allem auch aufgrund der gestiegenen relativen Bedeutung von Konsumentenmärkten in den Ländern des Globalen Südens (Horner und Nadvi 2018). Es kam aber zu keiner allgemeinen Deglobalisierung, und auch nach 2008/09 blieben die globalen Produktionsnetzwerke von zentraler Bedeutung.

## Treiber der räumlichen Restrukturierung

Um die „Slowbalization“ und den verstärkten Trend zur Regionalisierung zu erklären, werden zumeist fünf zentrale Treiber genannt. Es ist allerdings wichtig, einen differenzierten Blick auf diese Treiber zu werfen.

### a) Störungsanfälligkeit globaler Produktionsnetzwerke

Die Erschütterung der Lieferketten infolge der COVID-19-Pandemie war ein besonders drastisches Ereignis, steht aber in Kontinuität zu Störungen, die in den letzten Jahrzehnten deutlich zugenommen haben (vgl. Raza et al. 2021). Dies betrifft zum einen die Häufung von extremen Wetterereignissen und Naturkatastrophen, wie Überflutungen, Bränden und Erdbeben. Es wird erwartet, dass sich extreme Wetterereignisse infolge des Klimawandels vermehren, was die Störungsanfälligkeit von Lieferketten weiter erhöhen wird (ebd.). Zum anderen nehmen auch Interruptionen und Belastungen durch Handelskonflikte oder Cyberattacken zu. Lieferkettenunterbrechungen sind somit längst kein Ausnahmefall mehr, sondern stellen eine konstante Belastung des Welthandels dar. Die Befragten einer Studie des McKinsey Global Institute gaben an, dass es in ihrer Branche durchschnittlich alle 3,7 Jahre zu größeren Störungen kam, die einen Monat oder länger dauerten (Lund et al. 2020, S. 5), und solche Störungen haben gerade aufgrund der Vernetzung der Produktion schnell weltweite Auswirkungen. Während solche Disruptionen immer wieder Forderungen nach dem Rückbau globaler Lieferbeziehungen nach sich ziehen, wäre eine solche Antwort nicht unbedingt sinnvoll, und eine stärkere regionale Konzentration der Produktion könnte die Verwundbarkeit der Produktionsnetzwerke bei regional konzentrierten Schocks sogar erhöhen (Raza et al. 2021, S. 8).

### b) Gestiegene Arbeitskosten

Die eklatante Kluft zwischen den Arbeitskosten an den Standorten der globalen Exportproduktion im Globalen Süden und deren Zielmärkten im Globalen Norden besteht weiterhin, sie ist aber zuletzt vor allem in Bezug auf China zurückgegangen. Noch 2005 betrugen beispielsweise die durchschnittlichen Löhne der Industriearbieter*innen in China ein Zehntel von jenen in den USA. 2017 verdienten Arbeiter*innen in den USA „nur“ noch etwa drei Mal so viel wie ihre Kolleg*innen in China (Andersson et al. 2018). Im Vergleich mit typischen Nearshoring-Standorten wie der Türkei wird der schrumpfende Kostenunterschied noch deutlicher. 2017 verdienten Beschäftigte in der Industrie dort „nur“ noch etwa um die Hälfte höhere Einkommen als in China, während die Gehälter 2005 ungefähr fünf Mal so hoch waren (ebd.). Ein rein kostengetriebenes Offshoring nach China, der „factory of the world“, lohnt sich also immer weniger. Dies sollte allerdings nicht vorschnell als Anzeichen für ein Ende des globalen Sourcing im Allgemeinen und der Rolle Chinas als Knotenpunkt der Weltmarktproduktion im Besonderen gedeutet werden. Wie bereits in Abschnitt 2 argumentiert, sind neben den Arbeitskosten auch andere Standortfaktoren zentral, um interessant für Investitionen und Aufträge zu sein, allen voran Qualität, Lieferzeiten und Flexibilität. Darüber hinaus produziert China – sowie einige andere klassische Exportzentren – mittlerweile nicht nur billig, sondern eine große Bandbreite an Produkten von mittlerer bis teils auch hoher Komplexität, und viele Unternehmen sind in regionale Entwicklungs- und Fertigungscluster eingebunden (Butollo 2015). Das oftmals von Wirtschaft und Politik proklamierte Ziel, die Chinazentriertheit im Sourcing zu überwinden, stellt sich unter diesen Umständen als schwer zu realisieren heraus. Eine Alternative stellen „billigere“ Standorte wie Vietnam, Bangladesch, Kambodscha, Myanmar oder auch Afrika dar. Wirtschaftliche Aufholprozesse und Arbeitskämpfe führen aber auch in manchen dieser Länder zu steigenden Kosten, und sie können mit China vor allem auch hinsichtlich anderer Faktoren (wie z. B. der Fähigkeiten und Kapazitäten, dem Spektrum der Leistungen, der Qualität und der Einbindung in lokale Cluster) nicht konkurrieren.

### c) Digitalisierung der industriellen Produktion

Die Mutmaßung, dass die Digitalisierung der Fertigung eine Rückverlagerung der Produktion in den Globalen Norden begünstigen könnte, stützt sich vor allem auf zwei Annahmen (vgl. Kinkel 2020): Zum einen könnten die Arbeitskostenunterschiede infolge einer weitreichenden Automatisierung irrelevant werden. Zum anderen könnte eine Ansiedlung von Produktionsstandorten in unmittelbarer Nähe zu den jeweiligen Konsument*innenmärkten Konkurrenzvorteile bieten, weil im Zuge der Digitalisierung („Industrie 4.0“) eine flexiblere und kund*innengerechtere Fertigung möglich und immer relevanter werde. Solche Einschätzungen bleiben jedoch meist recht abstrakt, weil Reibungsverluste und gegenläufige Effekte der Digitalisierung vernachlässigt werden (vgl. Butollo 2020). Viele Interpretationen übernehmen unkritisch das Narrativ einer vierten industriellen Revolution, das technologiefixierte Fehlannahmen über Substitutionseffekte und Produktivitätssteigerungen enthält. Zudem wird meist nicht beachtet, dass Produktivitätssteigerungen nicht nur in den Ländern des Globalen Nordens zu verzeichnen sind. Insbesondere die nachholende Automatisierung an Schlüsselstandorten in China und Osteuropa (Butollo und Lüthje 2017; Schwarz-Kocher et al. 2019) führt eher zu einem erhöhten Verlagerungsdruck, da diese Standorte nun niedrige Kosten mit avancierter Produktionstechnik vereinen können. Schließlich stellen neue digitale Technologien, wie es bereits früher bei der Einführung der EDV und des Internets der Fall war, wichtige Mittel für die Ermöglichung sowie die bessere Koordination und Kontrolle von grenzüberschreitenden Produktionsprozessen dar. Der technologische Wandel kann also nicht auf einen einzigen Trend in Richtung Re- oder Nearshoring reduziert werden.

### d) Reorientierung der Handels- und Industriepolitik

Schon seit der Finanz- und Wirtschaftskrise 2008/09 ist in den USA und Europa eine Renaissance der strategischen Industriepolitik zu beobachten. Wichtige Gründe dafür sind geopolitische und -ökonomische Verschiebungen und das erklärte Ziel, die technologische Vorherrschaft der USA und der EU vor allem gegenüber China zu verteidigen. Die Wachstumspotenziale in technologieintensiven Feldern wie z. B. bei „grünen“ oder digitalen Innovationen ziehen diesbezüglich besondere Aufmerksamkeit auf sich (Eder und Schneider 2018; Rodrik 2008). In den USA gab es seit dem Amtsantritt von Trump eine Verschiebung hin zu einer stärker protektionistischen und neo-merkantilistischen Orientierung (Helleiner 2019). Diese Neuausrichtung wird weitgehend unter Präsident Biden fortgeführt und setzt u.a. auch auf das Reshoring durch Förderprogramme in strategisch wichtigen Sektoren. Dies geschieht besonders im Bereich der Hochtechnologieproduktion und ist im Kontext des verschärften Wettbewerbs mit China und des Handelskriegs zwischen den USA und China zu sehen (Raza et al. 2021). Im Zuge des Handelskriegs vervielfachten sich die Zölle auf chinesische Produkte um mehr als das Sechsfache von im Durchschnitt etwa 3 % im ersten Quartal 2018 auf fast 20 % im Jahr 2020.^2^ Diese Zölle betreffen zwei Drittel aller chinesischen Exporte in die USA, beinhalten aber auch Produktausnahmen (Brown 2021). In der EU ist diese Verschiebung der strategischen Ausrichtung noch nicht so deutlich zu sehen, aber Programme wie die neue EU-Industriestrategie (2020, aktualisiert 2021), die Europäische Digitalstrategie (2020) und der European Green Deal (2019) beinhalten zumindest die Möglichkeit einer umfangreichen industriepolitischen Neuausrichtung (Schlager und Soder 2020). Zwar können interventionistische Industriepolitiken zentrale Treiber für eine Regionalisierung der Produktion sein, aber zumindest vor der COVID-19-Pandemie gab es vor allem in der EU noch kaum konkrete Maßnahmen, was die Förderung von Near- oder Reshoring betrifft (siehe dazu Raza et al. 2021).

### e) Politische Reaktionen auf die Klima- und Umweltkrise

Im Kontext dieser Politikverschiebungen und der Formierung der neuen Europäischen Kommission hat auch die Klima- und Umweltkrise an Bedeutung gewonnen. Vor allem der Ende 2019 verabschiedete European Green Deal ist ein instruktives Beispiel für eine „grüne“ Industriepolitik (Europäische Kommission 2019). Neben der Realisierung von klimapolitischen Zielen soll der Green Deal v.a. dazu beitragen, Wettbewerbsfähigkeit, Innovation, Wachstum und Beschäftigung in Europa zu fördern und somit die Position Europas in der globalen Ökonomie zu stärken. Allerdings ist die Finanzierung dieser Vorhaben noch nicht geregelt (Schlager et al. 2020). Zudem setzt der Green Deal vor allem auf technologiefokussierte Effizienzstrategien. Konsistenzstrategien, die auf das Schließen von stofflichen und energetischen Kreisläufen abzielen, spielen zwar durch den Fokus auf die Kreislaufwirtschaft eine gewisse Rolle (Pakete zu Kreislaufwirtschaft 2015 und 2020), aber Suffizienzstrategien, die zu einer grundlegenden Änderung von (globalen) Produktions- und Konsummustern vor allem im Globalen Norden führen, fehlen ebenso wie eine globale Perspektive (Raza 2020b). Eine geografische Verkürzung von globalen Produktionsnetzwerken könnte durchaus Teil von „grünen“ Industriepolitiken sein; der Green Deal enthält jedoch keine direkten Maßnahmen zur Förderung von Nearshoring oder der Regionalisierung. Eine ernsthafte Bepreisung von CO_2_-Emissionen und der geplante CO_2_-Grenzausgleich würden die globale Produktion aber indirekt verteuern.

## Die Anatomie des COVID-19-Einbruchs

Zu dem skizzierten Bündel aus ökonomischen und politischen Veränderungen, die eine Diskussion um einen Rückbau globaler Produktionsnetzwerke befördert haben, kam 2020 die COVID-19-Pandemie mit ihren zum Teil massiven wirtschaftlichen Erschütterungen. Sie wird vielfach als möglicher Auslöser einer verstärkten Rückverlagerung der Produktion interpretiert, wobei meist keine genauere Analyse der differenzierten Ursachen für die wirtschaftlichen Einbrüche vorgenommen wird. Laut UNCTADstat ging der Wert des internationalen Handels (inklusive Dienstleistungen) und der ausländischen Direktinvestitionen im Jahr 2020 um 10,5 % bzw. 37 % zurück.^3^ Bei diesem Einbruch überlagerten sich angebots- und nachfrageseitige Ursachen (Baldwin und Freeman 2020), also das Ausbleiben von Rohstoffen, Komponenten und Endprodukten einerseits und der Rückgang und die Verschiebung der Nachfrage andererseits. Diese Ursachen wirkten sich aufgrund des sequenziellen Verlaufs der Pandemie in verschiedenen Regionen unterschiedlich bzw. zeitversetzt aus. In der ersten Phase standen Lieferengpässe aufgrund der weitgehenden Stilllegung der Produktion in China im Vordergrund, während in der zweiten Phase die wirtschaftlichen Verwerfungen vor allem auf die diversen Lockdowns in anderen Weltregionen zurückgingen. Im Folgenden skizzieren wir die vielfältigen pandemiebezogenen Ursachen der wirtschaftlichen Verwerfungen.

### a) Angebotsseitige Ursachen aufgrund der geografischen Konzentration von Produktionsstandorten

Lund et al. (2020, S. 11) identifizierten für das Jahr 2018 180 Produkte in globalen Produktionsnetzwerken, bei denen ein Land für mehr als 70 % der Exporte verantwortlich ist, was zu Flaschenhalseffekten führen kann. Diese umfassen diverse Rohstoffe, aber auch wichtige industriell produzierte Güter. Dies reflektiert zum Teil die besondere Rolle Chinas als Produktionsstätte für Automobilkomponenten, Elektronikprodukte, Bekleidung, Pharmazeutika und medizinische Schutzausrüstung. Infolge der Pandemie in China kam es aufgrund dieser hohen Konzentration zu globalen Engpässen u.a. bei wichtigen Medikamenten (Gereffi 2020; Raza et al. 2021). Auch die Lieferausfälle im Bereich der Halbleiterchips, die seit über zwei Jahren zu empfindlichen Stockungen der Produktion in verschiedenen Sektoren führen, ist zum Teil ein Resultat der enormen Konzentration der Chipproduktion an wenigen Standorten. Diese und andere angebotsseitige Einbrüche verstärkten die Wahrnehmung einer übermäßigen wirtschaftlichen Abhängigkeit von China und gaben Forderungen nach einem Re- und Nearshoring Auftrieb. Obwohl medial die Abhängigkeit von chinesischen Unternehmen im Vordergrund stand, waren die angebotsseitigen Probleme nicht nur durch Abhängigkeiten von der Produktion in Asien, sondern auch durch Störungen der intraregionalen Produktionsnetzwerke in Europa und Nordamerika bedingt. Der Lockdown in Italien führte beispielsweise zum Ausbleiben von Lieferungen wichtiger Komponenten in der Automobilindustrie (Buchenau 2020).

### b) Angebotsseitige Ursachen aufgrund von Just-in-time-Produktion in globalen Produktionsnetzwerken

Mit der geografischen Verschiebung der Schwerpunkte der Pandemie wurde die allgemeine Vulnerabilität vieler Produktionsnetzwerke sichtbar, die nicht primär mit deren geografischer Struktur, sondern mit der Just-in-time-Produktion und dem Paradigma flexibler Fertigung zusammenhängt. Ein Teilaspekt dieser Managementorientierung ist die verbreitete Praxis des „single sourcing“ – der Konzentration der Lieferkette auf einige Schlüssellieferanten –, wodurch Transportkosten und Lieferzeiten reduziert, die Flexibilität erhöht und Skaleneffekte erzielt werden können (Petersen 2020). Diese Managementstrategie macht jedoch Produktionsnetzwerke verletzlicher gegenüber Schocks und reduziert ihre Resilienz, unabhängig davon, ob sie global oder regional organisiert sind.

### c) Allgemeine Produktionsstillstände aufgrund von Shutdowns und Nachfrageausfällen

Obwohl die Pandemie die Krisenanfälligkeit von Produktionsnetzwerken in vielen Branchen offenbart hat, gingen einige Produktionsstillstände nicht auf Lieferprobleme bei Vorprodukten zurück. Einer Untersuchung über die Automobilindustrie zufolge war „der durch die Pandemie ausgelöste Shutdown des Betriebs die mit Abstand am häufigsten genannte Ursache für die Störung“ (Frieske und Stieler 2020, S. 31; Hervorheb. weggel.). Im Lauf der Zeit stabilisierten sich viele globale Produktionsnetzwerke zusehends, und die nachfrageseitigen Effekte traten immer stärker zum Vorschein (Grömling 2020). Nachfrageausfälle führten in einigen Sektoren sowie bei einigen allgemeinen Konsumgütern zu Produktionsreduktionen und -stillständen. Die diversen Produktkategorien und Produktionsnetzwerke waren aber sehr unterschiedlich betroffen, je nachdem, ob die Umsätze zurückgingen, stabil blieben (z. B. bei Grundnahrungsmitteln) oder sogar anstiegen (z. B. bei medizinischer Schutzausrüstung oder Desinfektionsmitteln).

### d) Lieferengpässe aufgrund sprunghaft gestiegener Nachfrage

Die Pandemie führte also auch zu einer Veränderung der Nachfragestruktur, und bestimmte Güter und Leistungen wurden verstärkt benötigt, was Engpässe nach sich ziehen konnte. Dies betraf zunächst vor allem Atemschutzmasken und Beatmungsgeräte für Intensivstationen, aber auch Medikamente. Die Engpässe waren nicht primär durch die starke Konzentration der Produktion v.a. in China bedingt. Europa ist unter normalen Umständen z. B. ein Nettoexporteur im Bereich der Medizintechnik und der medizinischen Schutzausrüstung (Gereffi 2020). Die eigentliche Ursache für die Engpässe lag vielmehr im sprunghaften und nicht vorherzusehenden Anstieg der globalen Nachfrage nach einem zuvor nur in spezifischen Bereichen verwendeten Produkt. Sogar in China, wo weltweit die mit Abstand größten Produktionskapazitäten bestehen, kam es im Verlauf der Pandemie zu Engpässen bei der Lieferung von Atemschutzmasken, und es mussten Presseberichten zufolge fast 2 Milliarden Masken importiert werden (OECD 2020). Im Laufe der Pandemie kam es auch zu erheblichen Nachfragesteigerungen bei einer Reihe von allgemeinen Konsumgütern wie Möbeln und Elektronikprodukten. Angebot und Nachfrage klafften auch in der strategisch wichtigen Halbleiterproduktion auseinander. Der Mangel an wichtigen Chips ist nicht nur durch angebotsseitige Störungen bedingt, sondern hängt auch mit dem starken Anstieg der Nachfrage zusammen. Aus diesem Grund werden derzeit die Produktionskapazitäten weltweit ausgebaut, was aber aufgrund des langzyklischen Charakters dieser Branche kaum zu einer kurzfristigen Entspannung der Lage führt (Rapp und Möbert 2022).

### e) Chronische Störungen der Handelswege und steigende Transportkosten

Pandemiebedingte Störungen belasteten insbesondere den Seehandel nachhaltig. Die temporäre Schließung oder Minderauslastung von Seehäfen im Zuge von Lockdowns, die im Verlauf der Pandemie in verschiedenen Regionen vorkamen, führten immer wieder zu punktuellen Unterbrechungen der Lieferketten. Eine völlige Normalisierung nach solchen Unterbrechungen, zu denen auch die Havarie eines Frachters im Suezkanal gehörte, erfolgte erst nach Wochen und Monaten, da jede Unterbrechung weitreichende Folgewirkungen auf die Handelsrouten hat: Schiffe stauen sich in den Häfen und können nicht eingesetzt werden, Container stranden an Umschlagplätzen an der Küste und im Landesinneren etc. (Kunst 2021; Buss et al. 2022, S. 51). Die deutlich gestiegene Nachfrage nach Konsumgütern im Jahr 2021 erschwerte die Normalisierung umso mehr, da die Seefrachtkapazitäten nahezu vollständig ausgelastet waren und der Rückstau an Gütern unter diesen Bedingungen nicht abgetragen werden konnte. Aufgrund der großen Nachfrage kam es auch zu erheblichen Preissteigerungen, vor allem für kurzfristige Transportslots, die gegenwärtig einen wesentlichen Inflationstreiber darstellen. Ein IMF-Bericht schätzt, dass sich zwischen dem Ausbruch der Pandemie und Oktober 2021 die Transportkosten für die Verschiffung von Massenwaren verdreifacht haben. Dies wird insbesondere auf die Containerknappheit und limitierte Hafenkapazitäten in Kombination mit verstärkter Nachfrage zurückgeführt (Carriere-Swallow et al. 2022, S. 6).

### f) Interventionen durch handelspolitische Beschränkungen

Fast 90 Länder führten mehr als 284 zeitliche Restriktionen bei Exporten seit dem Ausbruch der COVID-19-Pandemie ein, darunter auch Deutschland, die EU-Kommission und Japan.^4^ Solche handelspolitischen Eingriffe bestanden beispielsweise darin, dass China den Export von Schutzmasken der in China produzierenden amerikanischen Firma 3M untersagte, während die US-Regierung verlangte, dass die in den USA produzierten Masken von 3M nicht nach Kanada oder Lateinamerika exportiert werden (Gereffi 2020). Auch innerhalb der EU kam es phasenweise zu nationalen Exportverboten. Diese Verbote betrafen auch Produkte wie Nahrungsmittel und Toilettenpapier. Fast alle Länder in der OECD und einige Schwellenländer haben jedoch auch die Entwicklung oder Expansion von lokalen Produktionskapazitäten für die Produktion von Personal Protective Equipment (PPE) und PCR-Tests unterstützt (Raza et al. 2021). In China wurde die Produktion von Gesichtsmasken beispielsweise schon im Februar 2020 von 20 Millionen auf 110 Millionen Stück pro Tag hochgefahren, und im Sommer erreichte die Produktion 200 Millionen pro Tag. Die Produktion von PCR-Tests stieg von nahezu Null auf 2,6 Millionen pro Tag bis Mitte März 2020 (Duchâtel et al. 2020). Diese Produktion wurde im Inland verwendet, aber zum Teil auch exportiert.

Dieser differenzierende Blick auf die vielfältigen Ursachen der Unterbrechungen und Stockungen in den Produktionsnetzwerken relativiert die verbreitete Wahrnehmung, dass die globale Struktur der Produktionsnetzwerke im Allgemeinen und die Abhängigkeit von China im Besonderen die ausschlaggebenden Gründe für die wirtschaftlichen Verwerfungen infolge der Pandemie waren. Im Zuge der geografischen Ausbreitung und zeitlichen Sukzession der Pandemie traten auch intraregionale Störungen und massive Nachfrageverschiebungen als wesentliche Mitursachen für die Schwierigkeiten in unterschiedlichen Sektoren zutage. Seit 2021 stehen wiederum Kapazitätsengpässe sowie die chronische Überlastung des Seehandels und steigende Transportkosten im Vordergrund. Weiter anhaltende Störungen und Kostensteigerungen beim Transport werfen freilich Fragen über die Zukunftsfähigkeit der globalisierten Produktion und insbesondere des störungsanfälligen Just-in-time-Produktionsmodells auf.

## COVID-19 als Katalysator einer geografischen Neuordnung?

Die skizierten Störungen in globalen Produktionsnetzwerken im Zusammenhang mit der COVID-19-Pandemie haben Unternehmen und Politik für die Probleme durch Abhängigkeiten von globalen Zulieferfirmen und -regionen in bestimmten Produktgruppen sensibilisiert und zu verstärkten Debatten über die „Resilienz“ von globalen Produktionsnetzwerken geführt (Raza et al. 2021), worunter die Adaptionsfähigkeit sowie die Absicherung bestehender wirtschaftlicher Interaktionen verstanden wird. Befragungen von Unternehmen weisen auf die verstärkte Absicht hin, der Absicherung von Lieferketten einen höheren Stellenwert gegenüber bloßen Kostenfragen einzuräumen (Buchenau und Fröndhoff 2020). Eine globale Umfrage der Unternehmensberatung Ernst und Young unter Führungskräften aus vierzehn Industriebranchen im April 2020 zeigte, dass 83 % der Befragten auch ein Re- oder Nearshoring der Produktion in Erwägung zogen (Teigland et al. 2020).

Angesichts solcher Umfragen wurden zum Teil überschwängliche Erwartungen in Bezug auf einen Rück- oder Umbau der globalen Wertschöpfung formuliert (Haass 2020; Irwin 2020). Diese könnten sich aber als Trugschluss erweisen. Entsprechende Erwartungen wurden bereits nach der Finanz- und Wirtschaftskrise 2008/09 und auch nach der Reaktorkatastrophe von Fukushima 2011 gehegt. Ein nachhaltiger Rückbau der globalen Produktion und des Sourcing blieb jedoch aus. Die wechselseitige Abhängigkeit der Ökonomien hat sich seitdem sogar vergrößert, und insbesondere die Rolle Chinas ist noch bedeutsamer geworden (Baldwin und Freeman 2020). Die Länder des Globalen Südens unterliegen zudem gerade aufgrund der Pandemie strukturellen Zwängen, ihre Integration in globale Produktionsnetzwerke zu erhöhen, da die Kapitalabflüsse und die Auslandsverschuldung zugenommen haben (UNCTAD 2021). Aber auch unter den globalen Führungskräften hat sich im Laufe des Jahres 2020 die Stimmung bereits wieder gedreht: Bei einer späteren Umfrage von Ernst und Young im Oktober 2020 haben nur noch 37 % der Befragten Re- oder Nearshoring in Erwägung gezogen (Teigland et al. 2020).

Auch wenn die COVID-19-Pandemie aufgrund ihres globalen und zeitlichen Ausmaßes und der lange anhaltenden Folgewirkungen anders gelagert ist als punktuelle Schocks oder Krisen, wirken erhebliche Beharrungskräfte grundlegenderen Veränderungen entgegen. Ursächlich dafür ist nicht nur die ungebrochene Orientierung auf Just-in-time-Produktion und kurzfristige Effizienzgewinne, sondern sind auch die Pfadabhängigkeiten der bestehenden internationalen Arbeitsteilung und die Ballung von Produktionskapazitäten und -kompetenzen in nur schwer zu substituierenden Clustern. Zugleich besteht laut einer Studie des IFO-Instituts eine wirkliche Abhängigkeit von schwer zu ersetzenden Zulieferern nur bei ca. 5 % der Vorprodukte. Davon entfällt wiederum nur ein Bruchteil auf Zulieferer in Übersee. Im Fall von China geht es z. B. vor allem um Fahrradrahmen, Magnete und Ziergegenstände (Flach et al. 2021, S. 14 ff.). Es ist also nur sehr vereinzelt und für spezifische Produktgruppen mit einem marktgetriebenen Re- oder Nearshoring zu rechnen, was aber durch anhaltende Störungen im Seeverkehr und weiter steigende Transportkosten verstärkt werden könnte.

Entsprechend ist bislang, aller Rhetorik zum Trotz, keine konsequente geografische Reorientierung der Investitions- und Beschaffungsstrategien von Leitunternehmen zu erkennen. Stattdessen werden alternative resilienzorientierte Strategien verfolgt. In einer aktuellen Studie zum Thema heißt es: „Unabhängig vom Sektor plant ein Großteil der Unternehmen künftig bei der Beschaffung besser zu diversifizieren, die Lagerhaltung zu verstärken und die Lieferketten besser zu überwachen. Reshoring […], Nearshoring oder Insourcing werden hingegen relativ selten genannt und meist von nicht mehr als jedem zehnten Unternehmen in Betracht gezogen.“ (ebd., S. VIII; Hervorheb. weggel.) Bestrebungen zu einem intensiveren Monitoring, einer eingehenden Prüfung von Lieferanten („Due Diligence“) und einer Stärkung von logistischen Infrastrukturen haben nichts mit einem Rückbau, sondern vor allem mit einer Absicherung globaler Produktionsnetzwerke zu tun.^5^ Wie auch die Sektorstudien im folgenden Abschnitt zeigen, scheint eine Antwort von Leitunternehmen ein verstärkter Fokus auf Flexibilität und die Verwendung von digitalen Technologien zu sein – und kein grundsätzlicher Rückbau der Just-in-time-Produktion. Die zunehmende Diversifizierung von Produktionsnetzwerken, die dem Ziel folgt, lokal begrenzten Schocks besser ausweichen zu können, steht zudem in einem Spannungsverhältnis zu Strategien einer intraregionalen Konzentration durch Rückverlagerungen. Eine verstärkte Diversifizierung könnte außerdem das Machtgefüge weiter zugunsten von Leitunternehmen verschieben, da diese dann eine höhere Anzahl von Zulieferfirmen noch leichter gegeneinander ausspielen können.

Solche Verteilungskonflikte innerhalb der Produktionsnetzwerke kommen bei der Debatte um Reshoring generell zu kurz. Angesichts der großen Machtasymmetrien zwischen Leitunternehmen und Zulieferern ist es wahrscheinlich, dass schlussendlich die Zulieferfirmen die Kosten von höherer Lagerhaltung, Redundanzen und Transport zu tragen haben. Die Lagerhaltung war ja bereits vor COVID-19 im Rahmen des Just-in-time-Paradigmas nicht ganz verschwunden, sondern wurde von den Leitunternehmen an die Zulieferfirmen abgegeben, die mit den Kosten und Risiken umzugehen hatten. Schon zu Beginn der Pandemie haben die Leitunternehmen außerdem versucht, die Kosten des Lockdown-bedingten Nachfragerückgangs auf ihre Zulieferer abzuwälzen, indem sie Aufträge kündigten sowie ausstehende Rechnungen nicht, nur partiell oder zu spät beglichen (z. B. in der Bekleidungsindustrie: McNamara 2020). Die Konflikte um die Verteilung von Kosten und Risiken und die dahinterliegenden Machtasymmetrien erschweren eine Umorientierung in den Sourcing-Strategien, da Maßnahmen, die aus systemischer Sicht die Resilienz erhöhen könnten, in Konflikt mit den Eigeninteressen von Leitunternehmen, Zulieferfirmen oder Logistikern stehen können.

Relevanter für die geografische Struktur der Produktion in der (Post‑)COVID-19-Phase sind die politischen Initiativen zur Reorganisation von Produktion und Handel. Der Trend hin zu stärker strategischen Handels- und Industriepolitiken gewann in den USA und der EU schon vor der Pandemie vor allem aufgrund von geoökonomischen und -politischen Motivationen an Bedeutung, wurde aber durch COVID-19 verstärkt und um das Thema Versorgungssicherheit bei kritischen Gütern und Dienstleitungen erweitert (Dullien 2021). In der EU hat vor allem das Konzept der offenen strategischen Autonomie an Bedeutung gewonnen. Der Corona-Aufbauplan „NextGenerationEU“, den die Europäische Kommission als Reaktion auf die Pandemie aufgesetzt hat, fördert u.a. eine stärkere Präsenz von europäischen Unternehmen in digitalen Lieferketten^6^, und die „Strategic Investment Facility“ hat das Ziel, die Resilienz und strategische Autonomie bei zentralen Technologien und Lieferketten zu erhöhen und die Abhängigkeit von externen Zulieferern zu reduzieren^7^. Die EU-Rahmenregulierung „Foreign Investment Screening“, die ausländische (und v.a. chinesische) Übernahmen im High-Tech-Bereich erschweren soll, hat auch im Rahmen der COVID-19-Pandemie an Bedeutung gewonnen^8^. Die aktuelle EU-Industriestrategie beinhaltet des Weiteren die Überwachung strategischer Abhängigkeiten von einzelnen Produkten und die Definition von sechs strategisch wichtigen Bereichen – Rohstoffe, Batterien, pharmazeutische Wirkstoffe, Wasserstoff, Halbleiter und Cloud- und Spitzentechnologien. Das Förderprojekt „Important Projects of Common European Interest“ (IPEIS) soll europäische Wertschöpfungsketten durch transnationale Kooperationen und staatliche Beihilfen stärken, wovon bis jetzt aber überwiegend große Unternehmen in großen Mitgliedsstaaten profitieren (Berger und Soder 2021). Und wie der „US Chip Act“ soll der „European Chips Act“ die Wettbewerbsfähigkeit, technologische Führungsrolle und Resilienz bei Halbleitertechnologien und -anwendungen erhöhen.

Diese EU-Politiken repräsentieren einen Wandel in der politischen Orientierung und beinhalten eine stärkere interventionistische Rolle des Staates in der industriellen Transformation. Sie bleiben aber generell einer markt- und wettbewerbszentrierten Ausrichtung treu, die dem europäischen Integrationsprojekt zugrunde liegt (ebd.). Der Aufbau lokaler oder regionaler Produktionsnetzwerke spielt bis jetzt nur eine selektive produktspezifische Rolle, wie z. B. bei Mikroprozessoren und Elektrobatterien. Inwieweit sich die Absicht der Förderung lokaler oder regionaler Produktionsstrukturen in diesen und weiteren strategisch wichtigen Bereichen in voluminösen Förderprogrammen widerspiegeln wird und ob diese auch erfolgreich sind, ist bislang noch unklar. Dem politisch motivierten Re- und Nearshoring kommt aber potenziell eine bedeutendere Rolle zu als dem marktgetriebenen, durch Leitunternehmen veranlassten Nearshoring.

Wahrscheinlicher als eine umfassende Deglobalisierung ist ein verstärkte geopolitisch bedingte Grabenbildung zwischen rivalisierenden Weltregionen wie den USA und China und deren Freunden bzw. Verbündeten, was von der US Treasury Secretary Janet Yellen im April 2022 als „friend-shoring“ (danach auch als „ally-shoring“) bezeichnet wurde, also als Verlagerung in Länder, die unabhängig von ihrer geografischen Entfernung als Partner angesehen werden.^9^ Die geopolitische Zeitenwende infolge des russischen Angriffs auf die Ukraine verstärkt diese Tendenzen und könnte durchaus zu einer strategischen Neuausrichtung in den Geografien der Produktion und des Handels führen. Aber auch solche Bestrebungen werden limitiert durch gewachsene weltwirtschaftliche Entwicklungspfade und die mit ihnen verbundenen Interessen und Kräfteverhältnisse, wie zum Beispiel an der selektiven Ausgestaltung von Sanktionen gegen russische Importe zu sehen ist.

## Geografische Verschiebungen in der Automobil‑, Bekleidungs- und Elektronikindustrie

### COVID-19 als Schub intraregionaler Verlagerungen in der Automobilindustrie

Die COVID-19-Pandemie hatte starke Auswirkungen auf die globale Automobilindustrie. Ausschlaggebend dafür waren nicht nur Stockungen in der Lieferkette, sondern auch ein Einbruch der Nachfrage. Im ersten Halbjahr 2020 ging der Umsatz in den großen Absatzregionen China, USA und Europa um 28 % zurück, nur für Europa berechnet waren es sogar 39 % (VDA 2020). Allerdings scheinen zumindest die Endhersteller sehr gut aus der Krise gekommen zu sein. Daimler vermeldete beispielsweise trotz des Umsatzrückgangs (und auch infolge umfangreicher staatlicher Unterstützungsleistungen) für 2020 eine Steigerung der Gewinne um rund 50 % gegenüber 2019, und insgesamt scheint sich die Lage nach dem initialen Schock im Frühjahr 2020 recht schnell stabilisiert zu haben (ZEIT ONLINE 2021). Wie schon in der globalen Finanz- und Wirtschaftskrise 2008/09 wurden krisenbedingte Massenentlassungen in einigen Staaten der EU durch die Ausweitung des Kurzarbeitergeldes vermieden, was allerdings insbesondere in niedrigeren Einkommensgruppen mit empfindlichen Gehaltseinbußen verbunden ist. Dennoch wurden 2020 starke Stellenstreichungen an den globalen Standorten der Hersteller angekündigt und auch umgesetzt, bei denen es sich allerdings primär um die Fortsetzung allgemeiner Rationalisierungsmaßnahmen handelte, die schon vor der Pandemie begonnen hatten (Reimann 2020). Der Produktionsstillstand während des Lockdowns, der die Unternehmen sowieso vor die Herausforderung stellte, die Fertigung wieder hochzufahren, kann nun als Katalysator für Entscheidungen zu einer räumlichen Umstrukturierung der Produktionsnetzwerke wirken. Es ist jedoch eher eine weitere Auslagerung von Fertigungsschritten wahrscheinlich als eine Rückverlagerung von Produktionskapazitäten.

Die Automobilbranche ist ein anschauliches Beispiel für eine multiskalare Industrie, in der sich Prozesse globaler Fragmentierung und regionaler Integration überlagern. Obwohl die Fertigung einfacher Komponenten und eines wachsenden Anteils an elektronischen Elementen durchaus im globalen Maßstab geschieht, dominiert insgesamt eine intraregionale Produktionsstruktur, in der Endhersteller und weite Teile der Zulieferunternehmen in geografischer Nähe zu den wichtigsten Absatzmärkten angesiedelt sind (Sturgeon et al. 2008). Ausschlaggebend sind zum einen die Bedienung von regional spezifischen Kundenpräferenzen sowie Industrie- und Handelspolitiken, die aufgrund der hohen Bedeutung der Branche für Beschäftigung und Wertschöpfung auf eine weitgehende Lokalisierung der Produktion setzen. Kennzeichnend ist zum anderen eine intraregionale Arbeitsteilung, bei der Lohnkostenunterschiede eine große Rolle spielen. OEMs und Zulieferer haben in den letzten Jahrzehnten beispielsweise umfangreiche Produktionskapazitäten in Mexiko (Zielmarkt: USA) und Mittel- und Osteuropa (Zielmarkt: Zentraleuropa) aufgebaut.

Für die europäischen Produktionsstandorte ist die zunehmende Tendenz, die Produktion in räumlicher Nähe zu den Zielmärkten anzusiedeln, mit einem Verlust von Fertigungsanteilen verbunden. Weil die Produktion zunehmend intraregional stattfindet und sich der Schwerpunkt der globalen Nachfrage nach Asien verschoben hat, gingen Kapazitäten verloren, die vormals für den Export in anderen Weltregionen produziert wurden. So wurden im Jahr 2021 ca. drei Viertel der von deutschen Automobilherstellern produzierten PKWs im Ausland produziert.^10^ Branchenexpert*innen vermuten, dass die COVID-19-Krise diese Tendenz schon allein deswegen verstärken wird, weil die asiatischen Märkte aufgrund der schwachen Erholung der europäischen Märkte nun zum Wachstumsmotor der Automobilindustrie werden.

Eine weitere Ebene der Restrukturierung betrifft die anhaltende kostengetriebene Verlagerung des Wertschöpfungsschwerpunkts der europäischen Unternehmen im beschäftigungsintensiven Zulieferbereich. Der Anteil Mittel- und Osteuropas an den Arbeitsplätzen von Zulieferern in der Automobilindustrie in Europa hat sich zwischen 2008 und 2016 von knapp 40 auf ca. 48 % erhöht. Dies geschah vor allem auf Kosten der Beschäftigung in Westeuropa, aber nicht in Deutschland – dort ist der Beschäftigungsanteil relativ stabil geblieben (Frieske et al. 2019, S. 74). Dabei spielt auch eine industrielle Aufwertung der Standorte in Mittelosteuropa eine Rolle, die in technologischer Hinsicht keine große Differenz mehr zu den Werken deutscher Hersteller aufweisen und zunehmend auch Teile der Entwicklungsaufgaben übernehmen (Schröder und Krzywdzinski 2019).

Der Übergang zur Elektromobilität vertieft nun diese innereuropäische Arbeitsteilung, mit dramatischen Effekten für die Beschäftigung. Die OEMs nehmen mittlerweile keine großen Investitionen in die Weiterentwicklung des Verbrennungsmotors mehr vor. Dies schwächt aber, so die Einschätzung von Branchenexpert*innen, die Stellung der in Deutschland ansässigen Zuliefererstandorte in den beschäftigungsintensiven, mit dem herkömmlichen Antriebsstrang verknüpften Feldern. Einfache Anpassungen können auch durch die Entwicklungsabteilungen in Osteuropa geleistet werden, wo sie sich zugleich mit einer kostengünstigeren Fertigung verknüpfen lassen. Der aktuelle Rückzug aus der Verbrennungsmotortechnik führt daher zu einer Zunahme des Verlagerungsdrucks, und es gilt als unwahrscheinlich, dass neue Investitionen im Bereich der E‑Mobilität diese Beschäftigungsverluste kompensieren können (Frieske et al. 2019, S. 125 f.).

Aktuell überlagern sich diese strukturellen Umbrüche in der Automobilindustrie mit der COVID-19-Krise. Die Einbrüche auf den Absatzmärkten und die Unterbrechungen der Lieferketten verschärfen den ohnehin bestehenden Kostendruck. Nicht Reshoring, sondern eine Beschleunigung der Verlagerungstendenzen von Mittel- nach Osteuropa ist daher ebenso wahrscheinlich wie eine weitere Verschiebung der Markt- und Produktionsvolumina gen Asien. Unter dem Eindruck der Pandemie entstehen jedoch auch industriepolitische Initiativen, mit denen der Strukturwandel in den derzeitigen industriellen Kernregionen gefördert werden soll, um Fertigungskapazitäten zu halten. Die deutsche Regierung fördert beispielsweise im Rahmen ihres Konjunkturpakets Investitionen in Prozess- und Geschäftsmodellinnovationen sowie in die Elektromobilität und das autonome Fahren (BMWi 2021). Ob solche Ansätze die Verlagerungs- und Globalisierungstendenzen in der Branche aufhalten können, bleibt jedoch fraglich.

### COVID-19 als Verstärker von Restrukturierungen und Digitalisierung in der Bekleidungsindustrie

Der Bekleidungssektor wurde stark von der COVID-19-Pandemie und den damit verbundenen angebots- und nachfrageseitigen Störungen getroffen, welche weitreichende wirtschaftliche und soziale Auswirkungen für Zulieferfirmen und Arbeiter*innen nach sich zogen. Dies hängt damit zusammen, dass diese stark globalisierte Branche von einer flexiblen Just-in-time-Produktion, einer damit einhergehenden geringen Lagerhaltung und kurzen Lieferzeiten geprägt ist. Erste Unterbrechungen von Lieferketten durch Produktionsstopps traten zu Beginn des Jahres 2020 mit dem Ausbruch der Pandemie in China auf, dem global größten Textil- und Bekleidungsexporteur. Als sich die Pandemie weltweit ausbreitete, kam es in allen Regionen der Welt zu Unterbrechungen von Lieferketten, u.a. auch durch die Einschränkungen von Transportverbindungen und Logistikdienstleistungen. Danach folgten Nachfragerückgänge auf den Konsument*innenmärkten aufgrund der Lockdowns. Als Reaktion haben einige große Modemarken in der EU und den USA ihre Aufträge aufgrund von „force majeure“-Klauseln gekündigt, und sie verweigerten ihren Zulieferfirmen in den ersten Monaten der COVID-19-Pandemie die Bezahlung ausstehender Rechnungen in Höhe von bis zu 16 Milliarden US-Dollar (McNamara 2020). Zudem haben viele Leitunternehmen die Pandemie genutzt, um einen starken Druck auf die Preise auszuüben, Zahlungen zu verzögern und Verträge aufzuweichen. Eine Umfrage im Bekleidungssektor unter 75 Zulieferfirmen aus 15 Ländern ergab, dass sich die durchschnittlichen Zahlungsfristen von Leitunternehmen gegenüber Zulieferern von 43 auf 77 Tage erhöhten, bei einer gleichzeitigen Senkung der Preise um 12 % (Anner 2020).

Trotz der Arbeitsintensität im Bekleidungssektor und einer Verschiebung der Produktion in asiatische Produktionsländer in den letzten Dekaden spielen in dessen Geografie für die Endmärkte in der EU und den USA weiterhin regionale Zulieferländer eine Rolle: Zentral- und Osteuropa und Nordafrika für die EU sowie Mexiko und die zentralamerikanischen und karibischen Länder für die USA. Auschlaggebend dafür sind die Nähe zu den Endmärkten und die größere Flexibilität für Fast Fashion, aber auch regionale Handelsabkommen (Pickles et al. 2015). Ausgehend von dieser multiskalaren Ausgestaltung von Produktionsnetzwerken in der Bekleidungsindustrie ist zu erwarten, dass durch die COVID-19-Pandemie schon länger andauernde Restrukturierungs- und Verlagerungsprozesse verstärkt werden (Barrie 2020; ILO 2020). Diese wirken aber nur begrenzt in Richtung eines Re- bzw. Nearshoring.

Steigende Kosten vor allem in China, Probleme bezüglich der Einhaltung von Sozial- und Umweltstandards und Veränderungen in der Industriepolitik hin zu High-Tech-Sektoren in China und anderen asiatischen Ländern haben in den letzten zehn Jahren Verlagerungsprozesse innerhalb Asiens und weg von China vorangetrieben, die durch den Handelskrieg zwischen den USA und China beschleunigt und die COVID-19-Pandemie verstärkt wurden. Bei einer vom Beratungsunternehmen QIMA im Frühjahr 2020 durchgeführten Umfrage gaben weniger als die Hälfte der in der EU ansässigen Unternehmen und knapp zwei Drittel der asiatischen Käufer außerhalb Chinas an, kurzfristige Pläne für eine Restrukturierung ihres Zuliefernetzwerkes zu haben. In den USA gaben sogar fast 95 % der befragten Unternehmen an, aufgrund der COVID-19-Pandemie und des Handelskriegs mit China ihre Zulieferstruktur verändern zu wollen. Als bevorzugte neue Zulieferer werden in diesem Zusammenhang Vietnam (das von der Hälfte der Befragten präferiert wird) und Südasien genannt (30 % der Befragten äußerten eine Präferenz für Bangladesch oder Indien) (Barrie 2020). Der Standort China ist aber weiterhin von großer strategischer Relevanz aufgrund der Verfügbarkeit einer breiten Produktpalette, hoher Produktionsvolumina, der hohen Flexibilität der Produktion sowie seiner Bedeutung als Absatzmarkt (Langro und Lu 2021). Der chinesische Absatzmarkt spielt eine immer zentralere Rolle in den Wachstumsstrategien von Leitunternehmen, was die Bedeutung von chinesischen Zulieferfirmen und innerasiatischen Lieferketten bestärkt sowie zu einer Expansion von europäischen und amerikanischen Einzelhandelshäusern und Marken in China und anderen asiatischen Märkten führt (ILO 2020).

Ein Treiber von Re- und Nearshoring könnten aber die Störungen und Kostensteigerungen im Seehandel sein. Es ist noch nicht abzuschätzen, wie die Leitunternehmen damit umgehen werden. Zumindest eine Umfrage der Beratungsfirma McKinsey unter zehn Leitunternehmen aus den USA und Europa im Jahr 2021 legt nahe, dass 71 % der Chief Purchasing Officers (CPOs) erwarten, ihr Nearshoring zu erhöhen; 24 % erwarten sogar, das Reshoring ins Land ihres Head Quarters zu erhöhen (Hedrich et al. 2021, S. 21 f.). Die Realisierung solcher Vorhaben erscheint aber äußerst unrealistisch. Gegenwärtig gibt es erhebliche Kapazitätsengpässe, weshalb ein allgemeiner Rückzug aus dem Sourcing aus Übersee kurzfristig kaum möglich ist. Zunehmende Investitionen in die Türkei könnten aber von Bedeutung sein. Die Türkei ist das erste Mal in der letzten Dekade als Nearshore-Land in die Top 5 der Sourcing Locations gewählt worden (neben Bangladesch, Vietnam, Indonesien und China) (ebd., S. 20).

Auf der Ebene der Organisation von Produktionsnetzwerken wird erwartet, dass infolge der COVID-19-Pandemie die Konsolidierung auf der ersten Zulieferebene bei den sogenannten „core suppliers“ und vor allem den transnationalen asiatischen Produzenten aufgrund der gewachsenen Unsicherheiten weiter zunehmen wird. Diese „first tier“-Zulieferer können die ganze Lieferkette managen, ggf. auch die gesamte Produktion in einem Ort vertikal organisieren, aber auch flexibel auf unterschiedliche Produktionsländer zurückgreifen. Dies könnte zu einer Verkürzung und Vereinfachung von Lieferketten führen, nicht aber zu einem Rückbau ihrer globalen Reichweite. Die Position von kleineren Produzenten würde dadurch weiter geschwächt werden. Insolvenzen infolge der COVID-19-Krise verstärken diese Tendenzen (ILO 2020).

Automatisierung und Digitalisierung bei Textilproduktion, Logistik und Konsumtrends sowie beim Management von Qualität und Compliance gehören beim Geschäftsmodell des Fast Fashion zu den Kernstrategien von (Leit‑)Unternehmen des Bekleidungssektors (López et al. 2021). Da die Automatisierung der Produktion im Bekleidungssektor (im Gegensatz zum Textilsektor) trotz erster Experimente im Bereich der Robotik noch eine geringe Rolle spielt, gilt das vor allem für das Monitoring und die Steuerung der Lieferketten. Fast zwei Drittel der Befragten der bereits erwähnten QIMA-Umfrage gaben an, dass COVID-19 die Bemühungen ihres Unternehmens zur Digitalisierung der Lieferkette weiter verstärkt hat (Barrie 2020). Auch der Onlinehandel und die Nutzung der sozialen Medien zur Kundenbindung haben durch COVID-19 stark zugenommen. In Großbritannien ansässige Ultra-Fast-Fashion-Unternehmen wie Asos (Butler 2020), Missguided oder die Bohoo Group gehören zu den Krisengewinnern, obwohl ihr Umsatz noch weniger als 1 % der globalen Modeindustrie ausmacht. Sie sind ausschließlich auf den Onlinehandel spezialisiert und intensivieren das Fast-Fashion-Modell durch Lieferzeiten von unter zwei Wochen und das Angebot von bis zu 4500 neuen Produkten pro Woche (Wahnbaeck 2019). Maßstäbe setzen diese Unternehmen im Bereich der Digitalisierung der Lieferkette durch ihre datenbasierte reaktionsschnelle Produktion sowie die Vorwegnahme von Kund*innenpräferenzen (Camargo et al. 2020). Diese neuen Trends haben Auswirkungen auf die gesamte Bekleidungsindustrie. Fast-Fashion-Einzelhandelsketten wie Zara und H&M setzen nun ebenfalls verstärkt auf den Onlinehandel (López et al. 2021). Um die äußerst kurzen Lieferzeiten vor allem bei Produkteinführungen sicherzustellen, lassen die Ultra-Fast-Fashion-Unternehmen zum Teil direkt in der Nähe von Verteilerzentren in Großbritannien produzieren. Gleichzeitig zeigt ein Blick in die Gesamtheit der Lieferketten dieser Firmen, dass, trotz des Gebots, die Zielmärkte schnell zu beliefern, ein großer Teil der Produkte von Zulieferern in Zentral- und Osteuropa, aber auch in Asien gefertigt wird (ASOS 2021; Missguided 2021).

Ein weiterer wichtiger Trend ist der Anstieg von Nachhaltigkeitsregulierungen und -initiativen aufgrund der erheblichen Umweltbelastungen durch den globalen Textil- und Bekleidungssektor (Niinimäki et al. 2020). In den letzten Jahren lassen sich verstärkte Regulierungsinitiativen auf europäischer und nationalstaatlicher Ebene beobachten, wie der Green Deal und der Aktionsplan für Kreislaufwirtschaft, in denen der Textilsektor einen prioritären Sektor darstellt (Chua 2021), sowie die EU-Textilstrategie^11^. Vor dem Hintergrund dieser regulatorischen Änderungen, aber auch aufgrund der zunehmenden Wichtigkeit eines „nachhaltigen“ Images für Modeunternehmen, lässt sich in den letzten Jahren ein Anstieg an firmeneigenen Nachhaltigkeits- und Multi-Stakeholder-Initiativen beobachten. Höhere ökologische Anforderungen an die Zulieferfirmen und die dafür nötige Infrastruktur könnten Nearshoring-Prozesse verstärken. Aber auch die Firmen und Regierungen in den Produktionsländern in Asien und neue Zulieferländer wie Äthiopien investieren zunehmend in diesen Bereich (Jensen und Whitfield 2022).

### COVID-19 und die neue „China plus 1“-Strategie in der Elektronikindustrie

Die COVID-19-Pandemie hat in der ersten Phase aufgrund ihrer starken Konzentration in China deutliche Auswirkungen in der globalen Elektronikindustrie gezeitigt (IndustriALL 2020). Die damit einhergehenden Unterbrechungen der Lieferketten wirkten sich schnell auf andere Produktionsländer wie Malaysia und Indien aus. Darauf folgten nachfrageseitige Effekte aufgrund der weltweiten Lockdowns. Allerdings hatte die Pandemie zugleich einen Absatzschub zur Folge, weil Produkte für das Home Office und das Cloud Computing stärker nachgefragt wurden. Als Reaktion auf die Unterbrechungen in den Lieferketten wurde speziell im Elektroniksektor von unterschiedlicher Seite angekündigt, die Abhängigkeit von chinesischen Produktionsstandorten zu reduzieren und die Resilienz von Lieferketten zu erhöhen. Dies könnte die bereits bestehende „China plus 1“-Strategie von Leitunternehmen und Kontraktfertigern noch weiter verstärken. Eine solche Strategie der Diversifizierung über China hinaus wurde aufgrund steigender Arbeitskosten, Sorgen um den Schutz geistigen Eigentums und geopolitischer Veränderungen schon vor der Pandemie verfolgt (Patterson 2020), war aber schwierig zu realisieren.

Generell ist die Geografie der Produktion in der globalen Elektronikindustrie durch eine Vielzahl an Fragmentierungs- und Reintegrationsprozessen gekennzeichnet und ähnlich komplex wie in der Automobilindustrie. Ein wesentlicher Unterschied besteht in einer höheren Zahl an Endprodukten, deren Lieferketten sich teilweise stark unterscheiden. So ist etwa die Fertigung von Mobiltelefonen beim Weltmarktführer Samsung stark global konzentriert – rund die Hälfte aller Samsung-Mobiltelefone kommen aus dem eigenen Werk in Vietnam, von wo sie an regionale Hubs wie etwa das slowakische Samsung-Werk für das Fine-Tuning vor dem europäischen Vertrieb verteilt werden. Im Gegensatz dazu finden sich im Bereich der industriellen oder medizinischen Elektronik stärker dezentral ausgerichtete Produktionsprozesse, weil in diesem Produktsegment die an den Endmärkten ausgerichteten öffentlichen und privaten Regulierungen sowie zeitkritische Dienstleistungen wie Wartung und Reparatur eine größere Bedeutung haben (Hamrick und Bamber 2019). Durch die Digitalisierung wird diese Diversität noch erhöht, da elektronische Komponenten und digitale Technologien zunehmend Eingang in andere Industrien finden, wie z. B. die Elektromobilität, smarte Bekleidung und den Gesundheitssektor (Raj-Reichert 2018).

Ungeachtet dieser Diversität spielt China eine besondere Rolle als Gravitationszentrum der globalen Elektronikproduktion, was eine Folge von umfangreichen Produktionsauslagerungen an Kontraktfertiger wie Foxconn, Flextronics, Jabil oder Compal und Asustek ist (Lüthje et al. 2013). Die räumliche Konzentration der Fertigung in China ist schon längst nicht mehr rein kostenbedingt, sondern hängt mit der Existenz eines komplexen Ökosystems aus Entwicklungs- und Fertigungsprozessen eines breiten Spektrums von Komponenten zusammen. Mit den Investitionen in anderen Standorten wie Vietnam versuchen globale Produzenten und Leitunternehmen zu diversifizieren, kommen aber bislang nicht über eine Ergänzung ihrer China-zentrierten Produktionsnetzwerke hinaus (Pandit 2020). Ungeachtet der globalen Verschiebung in Richtung asiatischem Raum weisen die Produktionsnetzwerke der Elektronikindustrie, abhängig von Produkttyp und Fertigungsverfahren, eine bedeutende makro-regionale Komponente auf, insofern Standorte der Endmontage in räumlicher Nähe zu den zentralen Absatzmärkten errichtet werden – in Mittel- und Osteuropa für die zentralen Endmärkte in Europa und in Mexiko für die USA (Lüthje et al. 2013).

Aufgrund der COVID-19-Pandemie und der gestiegenen Aufmerksamkeit für die Abhängigkeit von der Elektronikfertigung in Asien sowie der schon länger zunehmenden geopolitischen Rivalitäten vor allem zwischen den USA und China, aber auch zwischen der EU und China könnten Initiativen zu einer geografischen Umorientierung des Sourcing an Durchschlagkraft gewinnen. Forderungen nach einer Rückverlagerung bzw. einem Wiederaufbau von industriellen Kapazitäten in der Elektronikindustrie in der EU und den USA haben daher eine deutliche geopolitische Dimension. Bei der Rivalität zwischen den USA und China geht es um die Führungsrolle bei Schlüsseltechnologien, z. B. im Bereich der Künstlichen Intelligenz oder des Internets der Dinge. Und da solche und andere Produkte die Grundlage für Kommunikationsinfrastrukturen und eine breite Palette von Produkten bilden, berührt die Frage der Geografie der Produktion auch die Frage der technologischen Souveränität angesichts zunehmender Handelskonflikte.

Die EU fördert vor diesem Hintergrund und im Zusammenhang mit dem Green Deal industriepolitische Initiativen zur Unterstützung strategisch wichtiger Wertschöpfungsketten. Seit 2014 schon gibt es eine öffentlich-private Partnerschaft zur Sicherung der europäischen Vorherrschaft im Bereich von elektronischen Komponenten (ECSEL) und seit 2020 das Nachfolgeprogramm Key Digital Technologies (KDT). Ob die hehren Ziele jedoch mit den eingesetzten Mitteln erreicht werden können – das Zehnjahresbudget von ECSEL beträgt lediglich 5 Mrd. Euro –, ist fraglich. Angesichts des umfangreichen Verlustes an industrieller Produktion in Europa und den USA und der Konzentration von Fertigungskapazitäten und Know-how in Asien in industriellen Clustern und Ökosystemen wären wohl größere Investitionen bzw. stärkere staatliche Eingriffe notwendig, um eine substanzielle Reindustrialisierung voranzutreiben (Beattie 2020; Thun et al. 2021). Und mit Blick auf die Elektronikindustrie stellt sich die grundsätzliche Frage, inwieweit die Relokalisierung der Produktion in einer Branche, die seit Jahrzehnten zu einem der am stärksten globalisierten Sektoren gehört, überhaupt kurzfristig gelingen kann.

## Schlussfolgerungen

Die kurzen Synopsen zu den aktuellen Restrukturierungen in drei wichtigen Wirtschaftssektoren zeigen, dass es voreilig wäre, von einer allgemeinen Tendenz eines Re- und Nearshoring oder gar einer Deglobalisierung infolge der COVID-19-Pandemie auszugehen. Die Strategien der Leitunternehmen zielen eher darauf ab, die Vorteile eines global ausgerichteten Sourcing und von regionalen Knotenpunkten der Fertigung mit einfachen Zugängen zu relevanten Zielmärkten zu kombinieren sowie das Paradigma der Just-in-time-Produktion aufrechtzuerhalten. In der ohnehin primär regional organisierten Automobilindustrie treiben die Folgen der Pandemie am ehesten ein vermehrtes Offshoring von Fertigungskapazitäten in grenznahe Standorte mit niedrigeren Kosten voran, was auch im Zusammenhang mit der Transformation zur Elektromobilität steht. In der Bekleidungsindustrie könnte das Ursachenbündel aus hohen Transportkosten, Ultra-Fast Fashion und höheren Umweltauflagen durchaus Tendenzen des Nearshoring befördern, ohne dass dies jedoch einen allgemeinen Rückbau des globalen Sourcing mit sich bringen würde. In der stark global strukturierten Elektronikindustrie gibt es zwar staatlich geförderte Versuche zur Wiederansiedlung von Produktionsstandorten für Schlüsselkomponenten. Es muss sich aber erst noch zeigen, ob diese Bemühungen von Erfolg gekrönt sein werden. Zudem zielen solche Initiativen nicht auf die Rückverlagerung des gesamten Produktions- und Handelsvolumens, sondern auf einige strategisch wichtige Komponenten. In allen drei Sektoren scheint die Antwort auf COVID-19 in einem verstärkten Fokus auf kurze Lieferzeiten, Flexibilität und die Verwendung von digitalen Technologien zu bestehen und nicht in einem grundsätzlichen Rückbau globaler Produktion.

Insgesamt überwiegt somit trotz der epochalen Erschütterung globaler Produktionsnetzwerke durch die Pandemie bei der sozialräumlichen Struktur der Weltwirtschaft eher Kontinuität als ein grundlegender Wandel. Allerdings hat das Thema der Resilienz, im Sinne der Versorgungssicherheit von Lieferketten, infolge von COVID-19 eine erhöhte Relevanz und Aufmerksamkeit erhalten. Dabei verbinden sich die durch die Pandemie angestoßenen Überlegungen mit bereits bestehenden handels- und industriepolitischen Bestrebungen in den USA und Europa, die Wettbewerbsfähigkeit und technologische Vorherrschaft in strategisch wichtigen Feldern zu behaupten bzw. zurückzugewinnen sowie die Abhängigkeiten vor allem gegenüber China zu reduzieren. In diesen Feldern ist eine vermehrte wirtschaftliche und politische Dynamik zu erwarten, wobei ihre Wirkungen eher punktuell bedeutsame Glieder in globalen Produktionsnetzwerken (z. B. Halbleiterchips, Elektrobatterien oder bestimmte medizinische Produkte und Medikamente) betreffen, als dass sie auf eine allgemeine geografische Reorientierung der Produktion hinausliefen. Die geopolitischen Konflikte nahmen durch den russischen Krieg gegen die Ukraine zu und verstärken Tendenzen zur Blockbildung; ob das zu einer politisch getriebenen Spaltung des Welthandels führt, muss sich aber erst zeigen.

Politische Ziele sind immer umkämpft und stehen in einem Spannungsverhältnis zu den jeweiligen Kräfteverhältnissen und den Pfadabhängigkeiten in einer multipolaren Weltordnung. Marktliberale Dogmen in der Handels- und Wettbewerbspolitik sind in der EU und auch in den nationalen Institutionen weiterhin stark verankert. Zudem decken sich die politischen Proklamationen oft nicht mit den Zielen der (Leit‑)Unternehmen verschiedener Branchen. Politische Projekte für eine umfassende geografische Neuorientierung müssten somit erhebliche Ressourcen mobilisieren und Konflikte in Kauf nehmen, was sich aus den bisherigen Stellungnahmen nicht ablesen lässt. Bei den Leitunternehmen könnten die mittelfristigen Folgen der Pandemie dazu beitragen, dass ihre Entscheidungen nicht resilienz-, sondern noch stärker kosten- und flexibilitätsorientiert ausfallen, also das neue Bewusstsein für die Fragilität globaler Produktionsnetzwerken schon bald wieder dem Business as Usual weicht.

Doch steckt in der Pandemie auch eine Chance, denn sie wirft fundamentalere Fragen nach der Zukunftsfähigkeit der derzeitigen ökonomischen Ordnung auf. Damit ist auch die Frage verbunden, unter welchen Umständen die Politik in Form einer strategischen Industrie- und Handelspolitik zum Treiber einer Restrukturierung globaler Produktionsnetzwerke und einer sozial-ökologischen Transformation werden könnte. Die notwendige Dekarbonisierung der Wirtschaft setzt eine Verkürzung von globalen Produktionsnetzwerken bzw. deren stärkere Regionalisierung voraus. Dies meint nicht Autarkie, sondern eine sektoral differenzierte Deglobalisierung von Produktionsnetzwerken. So wäre es etwa unmöglich und widersinnig zu versuchen, die Elektronikproduktion als Ganzes auf lokaler Ebene anzusiedeln. Hingegen könnte die Produktion von alltäglichen Bedarfsgütern (z. B. Lebensmittel, Bekleidung, Möbel) sowie von kritischen medizinischen oder pharmakologischen Produkten sehr wohl stärker auf regionaler oder lokaler Ebene stattfinden. Die Stärkung von lokalisierten Wirtschaftskreisläufen, die an den grundlegenden und universellen Bedürfnissen der Menschen ansetzen, steht im Zentrum von alternativen Entwicklungsstrategien wie dem Konzept der Foundational Economy^12^, das eine an gesellschaftlichen Zielsetzungen orientierte Reorganisation der öffentlichen Daseinsvorsorge (Wasser, Energie, Mobilität, Gesundheit, Bildung), der Produktion von Gütern und Dienstleistungen, die für das tägliche Überleben notwendig sind (Lebensmittel, Wohnen), sowie bestimmter Konsumgüter (Bekleidung, Möbel) vorsieht.

Für die Länder des Globalen Südens würden solche Strategien in der EU und den USA zu einer Reduktion ihrer Exporte führen, was aber auch Chancen für eine Refokussierung weg von primär exportorientierten Entwicklungsmodellen und einer einseitigen Orientierung auf den Weltmarkt bieten würde. Eine strategische und selektive Abkoppelung von globalen Produktionsnetzwerken und alternative Integrationsprojekte im Rahmen lokaler und regionaler Produktionsnetzwerke könnten dazu beitragen, die eigene industrielle Basis zu verbreitern und wirtschaftliche Unabhängigkeit zu stärken. Die Nutzung dieses Potenzials hängt aber nicht zuletzt von industriepolitischen Maßnahmen und vom politischen Handlungsspielraum in den Ländern des Globalen Südens ab, der durch derzeitige Handelspolitiken und damit verbundene Abkommen eingeschränkt wird.

Eine partielle Deglobalisierung im Zuge einer sozial-ökologischen Transformation sollte eine Maxime für die Rekonstruktion der Weltwirtschaft im Nachklang der COVID-19-Pandemie sein. Die staatliche Handels- und Industriepolitik, die nun wieder eine stärkere Beachtung findet, bietet einige Instrumente dafür, die allerdings deutliche Eingriffe in die Entscheidungsautonomie von (Leit‑)Unternehmen erfordern würden. Beihilfen für Unternehmen und industriepolitische Maßnahmen sollten beispielsweise an die Umsetzung von Klimaschutzmaßnahmen und die Einhaltung sozialer Zielsetzungen gekoppelt werden. Erforderlich ist aber auch eine Umorientierung der auf Exportüberschüsse ausgerichteten Handelspolitik sowie der auf globale Wettbewerbsfähigkeit und Standortvorteile ausgerichteten Industriepolitik. Dies bedarf einer neuen Generation fairer Handelsabkommen, die die verbindliche Einhaltung von sozialen und ökologischen Standards und die gerechte Verteilung der Wertschöpfungsanteile, Kosten und Risiken gewährleistet, einen politischen Handlungsspielraum für eigene Entwicklungsstrategien (auch innerhalb der EU) gewährt und die grundlegenden Bedürfnisse der Menschen im Globalen Norden und im Globalen Süden ins Zentrum stellt.^13^ Ein wichtiges Feld einer solchen auf Nachhaltigkeit und globale Gerechtigkeit zielenden weltwirtschaftlichen Neuordnung ist die Versorgung mit medizinischen Produkten, Medikamenten und Impfstoffen. Der politische Umgang mit der Pandemie hat diesbezüglich genau die großen Ungleichheiten vor Augen geführt, die es zu bekämpfen gilt.

## Footnotes

1 Das Sample umfasst drei Expert*innen aus Unternehmensverbänden, drei Vertreter*innen und Expert*innen der Automobil- sowie zwei der Elektronikindustrie. Für den Bekleidungssektor wurden vor allem Berichte aus Just Style, Sourcing Journal und Apparel Resources sowie Diskussionen beim Copenhagen Fashion Summit 2020 und 2021, bei der GFA CEO Agenda 2021 und beim Sourcing Journal Summit 2020 analysiert.

2 Die durchschnittlichen Zölle auf US-Importe erreichten in China im Jahr 2020 ebenfalls einen Wert von etwa 20 % (Brown 2021).

3 https://unctadstat.unctad.org/EN/. Zugegriffen: Febr. 2022.

4 https://www.wto.org/english/tratop_e/covid19_e/trade_related_goods_measure_e.htm. Zugegriffen: Mai 2021.

5 Der weltweit größte Logistiker DHL verfolgt beispielsweise mit seiner „Resilience360-Plattform“ das Ziel, Risiken wie „Naturkatastrophen, Cyberangriffe und sich schnell verändernde regulatorische Rahmenbedingungen“ schnell vorherzusehen und globale Lieferketten gegen Störungen zu schützen (DHL 2019).

6 https://europa.eu/next-generation-eu/index_de. Zugegriffen: Juli 2022.

7 https://www.consilium.europa.eu/de/policies/investment-plan/strategic-investments-fund/. Zugegriffen: Juli 2022.

8 https://ec.europa.eu/commission/presscorner/detail/de/ip_20_1867. Zugegriffen: Juli 2022.

9 https://www.atlanticcouncil.org/news/transcripts/transcript-us-treasury-secretary-janet-yellen-on-the-next-steps-for-russia-sanctions-and-friend-shoring-supply-chains/. Zugegriffen: Juli 2022.

10 https://www.vda.de/de/aktuelles/zahlen-und-daten/jahreszahlen/automobilproduktion. Zugegriffen: März 2022.

11 https://environment.ec.europa.eu/strategy/textiles-strategy_de. Zugegriffen: Juli 2022.

12 https://foundationaleconomy.com/.

13 https://www.anders-handeln.at/.
